# Inflammation-related biomarkers for intracardiac thrombosis in acute myocardial infarction: predictive value and mechanistic implications of NLR and LDL-C

**DOI:** 10.3389/fmed.2025.1643933

**Published:** 2025-08-05

**Authors:** Sijie Zhao, Zizhu Lian, Xiaoning Wang, Na Mei

**Affiliations:** ^1^Department of Hematology, The First Affiliated Hospital of Xi’an Jiao Tong University, Xi’an, China; ^2^Department of Cardiovascular Surgery, The First Affiliated Hospital of Xi’an Jiao Tong University, Xi’an, China; ^3^Interventional Operating Room, The First Affiliated Hospital of Xi’an Jiao Tong University, Xi’an, China

**Keywords:** acute myocardial infarction, intracardiac thrombosis, neutrophil-to-lymphocyte ratio, low-density lipoprotein cholesterol, inflammatory biomarkers, risk prediction

## Abstract

**Background:**

Inflammatory pathways critically contribute to the pathogenesis of intracardiac thrombosis (ICT) following acute myocardial infarction (AMI) patients. This study evaluated the predictive value of inflammation biomarkers for ICT.

**Methods:**

This retrospective case–control study included 8,999 AMI patients hospitalized at the First Affiliated Hospital of Xi’an Jiaotong University from January 2018 to December 2022, among whom 88 developed ICT. To address class imbalance, 891 non-ICT patients were randomly selected as controls using a 1:10 ratio. Inflammation-related biomarkers were screened using univariate and multivariate logistic regression, and a risk heatmap was generated based on key predictors.

**Results:**

Multivariate logistic regression identified elevated neutrophil-to-lymphocyte ratio (NLR), N-terminal pro–B-type natriuretic peptide (NT-proBNP), low-density lipoprotein cholesterol (LDL-C), and reduced albumin-to-globulin ratio (AGR) as independent risk factors for ICT. NLR demonstrated the highest discriminatory capacity, demonstrating superior predictive performance [receiver operating characteristic (ROC) curve, area under the curve (AUC) = 0.774, 95% confidence interval (CI): 0.724–0.823, *p* < 0.001] that persisted after full covariate adjustment, and remained significant after full adjustment [odds ratio (OR) = 2.54, 95% CI: 1.98–3.15, *p* = 0.002]. Integration of NLR and LDL-C into a sex-stratified risk stratification matrix significantly enhanced diagnostic accuracy (AUC = 0.838, 95% CI: 0.799–0.878).

**Conclusion:**

This study established NLR as a robust indicator for ICT assessment and presents a practical, visual risk heatmap that may facilitate personalized thromboprophylaxis in AMI management.

## 1 Introduction

Acute myocardial infarction (AMI) remains a leading global health burden, with a steadily increasing incidence worldwide (1). AMI initiates a complex pathophysiological cascade, including inflammation, oxidative stress, platelet activation, and thrombus formation. Among these, intracardiac thrombosis (ICT) represents a clinically significant and potentially life-threatening complication, as it can compromise cardiac function and precipitate heart failure or myocardial rupture (2). Dislodged thrombi may cause major embolic events—such as ischemic stroke, acute limb ischemia, or pulmonary embolism—substantially increasing morbidity and mortality (3–5). However, ICT is often subclinical and lacks overt symptoms. Its diagnosis depends heavily on imaging, which is limited by cost and equipment availability, making widespread screening impractical (6). These limitations underscore the need for alternative, cost-effective, and readily available biomarkers for early detection and risk stratification.

Recent studies have demonstrated that persistent low-grade inflammation plays a central role in the onset, progression, and complications of cardiovascular diseases (5). In AMI patients, inflammation not only destabilizes atherosclerotic plaques but also contributes to thrombogenesis by activating neutrophils, promoting platelet aggregation, and impairing endothelial function—key mechanisms in ICT development (7). The presence and intensity of chronic inflammation can be reflected by circulating inflammatory biomarkers (8). Among these, the neutrophil-to-lymphocyte ratio (NLR), a simple marker derived from routine blood counts, has emerged as a reliable predictor of cardiovascular outcomes and thrombotic risk (8, 9). NLR reflects the dynamic balance between innate and adaptive immunity, with elevated levels indicating systemic inflammation. A prospective study by Song et al. (10) confirmed that elevated NLR is associated with cause-specific mortality—including cardiovascular, respiratory, infectious, and renal diseases—highlighting its potential as a risk stratification tool in both public health and clinical settings (11).

In addition to NLR, several other routinely assessed serum biomarkers are known to correlate with thrombosis risk and myocardia function. These include markers of myocardial injury or dysfunction [e.g., N-terminal pro–B-type natriuretic peptide (NT-proBNP), creatine kinase-MB (CK-MB), and creatine kinase (CK)], lipid metabolism indicators [e.g., high-density lipoprotein cholesterol (HDL-C) and low-density lipoprotein cholesterol (LDL-C)], as well as composite indices such as the albumin-to-globulin ratio (AGR) and the atherogenic plasma index (API).

While inflammation is recognized as a key factor in AMI prognosis, the association between inflammatory markers and the risk of ICT remains insufficiently characterized. This study focuses on NLR and other accessible serum biomarkers to evaluate their predictive value for ICT in AMI patients and to propose a practical early risk stratification model to guide clinical management.

## 2 Methods

### 2.1 Study design and patients

This retrospective observational study was conducted at the First Affiliated Hospital of Xi’an Jiaotong University (No. 277, West Yanta Road, Xi’an, Shaanxi Province, 710061, China). Clinical data were retrospectively collected from patients diagnosed with acute myocardial infarction (AMI) between January 2018 and December 2022. AMI and ICT were diagnosed according to established criteria (12, 13), and treatment protocols adhered to the 2023 European Society of Cardiology guidelines for the management of acute coronary syndromes (14). Eligible patients included those with confirmed diagnoses of AMI, including both ST-elevation myocardial infarction and non-ST-elevation myocardial infarction. ICT was confirmed through echocardiographic imaging performed during hospitalization, encompassing thrombus detection in the left and right ventricles, atria, atrial appendages, apex, or other cardiac chambers (6, 15). All AMI patients underwent transthoracic echocardiography during admission as part of routine inpatient assessment, allowing for systematic detection of intracardiac thrombus. Standardized transthoracic echocardiography (TTE) was performed on all patients within 24 h of admission using with apical 4-chamber and subcostal views for thrombus screening.

Patients with missing data for the majority of laboratory parameters were excluded. Given the low incidence of ICT, a 1:10 random sampling strategy was employed to select non-ICT controls, thereby addressing extreme class imbalance and enhancing model robustness. To preserve the integrity of variable distribution and avoid masking the effects of potential risk factors, we did not perform matching during control selection. Instead, all relevant covariates were included in the multivariate regression models to adjust for confounding. To evaluate potential sampling bias, we additionally compared baseline characteristics between the included non-ICT controls and the excluded AMI patients without ICT; no significant differences were observed (Supplementary Table S1). All ICT cases included were first-time diagnoses, and patients with active infections within the preceding month were excluded to minimize confounding. For patients with partial laboratory data missing, multiple imputation was applied to preserve statistical power and optimize model discrimination.

**Figure 1:**
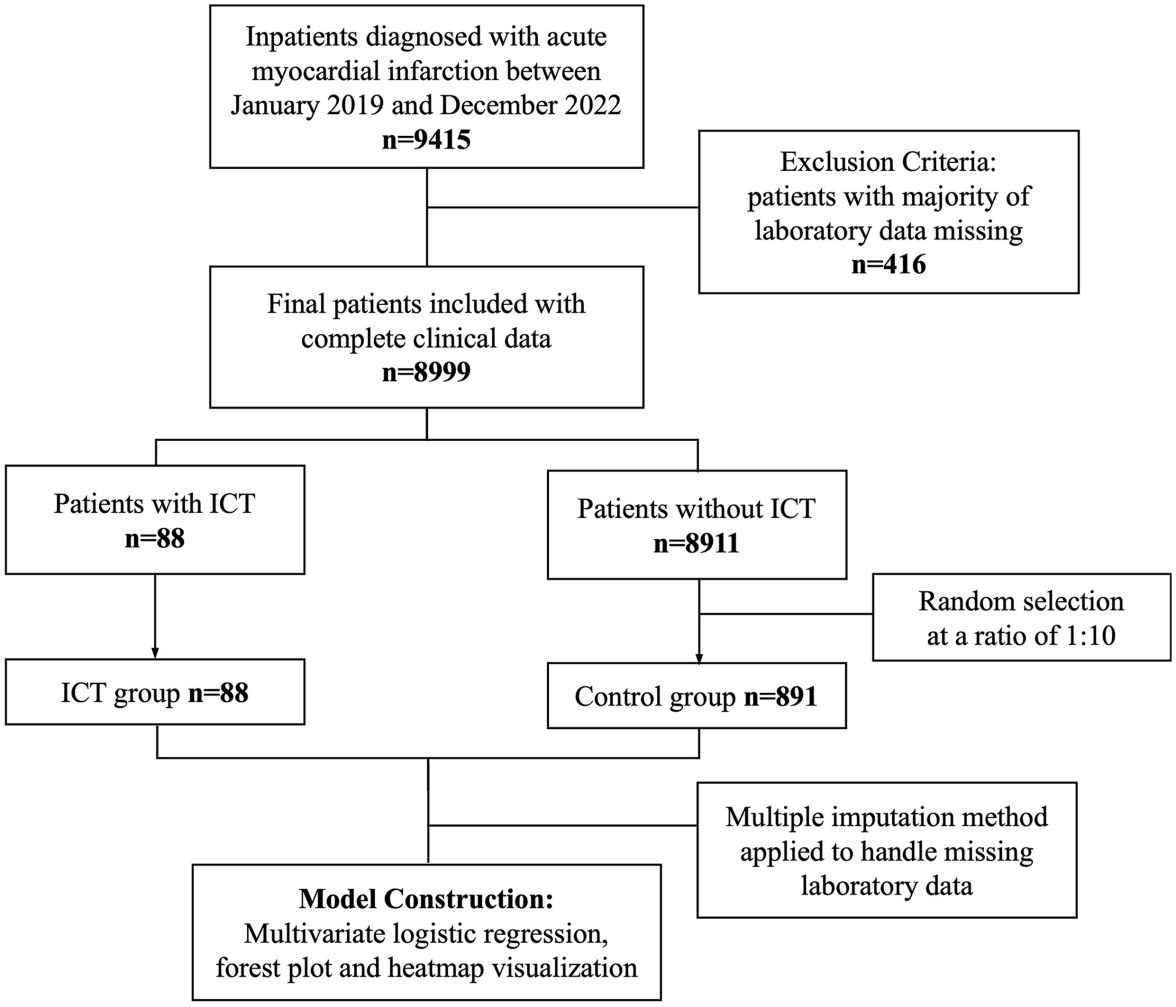
A flowchart of the study.

The study protocol conformed to the ethical standards of the Declaration of Helsinki (1975, revised in 2024) and was approved by the Institutional Review Board of Xi’an Jiaotong University (Approval No. XJTU1AF2025LSYY-410). As this study involved retrospective analysis of de-identified data obtained from the hospital’s biobank, the requirement for written informed consent was formally waived. All data handling complied with institutional protocols governing the secondary use of biomedical data. An overview of the study design and patient selection process is shown in Figure 1.

### 2.2 Data collection

Comprehensive baseline data were systematically collected at the time of hospital admission, encompassing demographic characteristics, pre-existing comorbidities, and heart failure status assessed via the Killip classification. Laboratory parameters were extracted from the hospital’s electronic medical records and included cardiac injury markers, complete blood count indices, absolute neutrophil count (ANC), absolute lymphocyte count (ALC), lipid profiles, hepatic and renal function tests, as well as serum electrolyte levels.

Inflammation-related composite biomarkers were calculated as follows: the neutrophil-to-lymphocyte ratio (NLR) was derived by dividing the neutrophil count by the lymphocyte count [NLR = neutrophil count (NEUT) / ALC], and the albumin-to-globulin ratio (AGR) was computed as serum albumin divided by globulin [AGR = ALB / total protein (TP) – albumin (ALB)]. All laboratory values were obtained upon admission to ensure consistency and minimize potential confounding due to treatment interventions.

### 2.3 Statistical analysis

Continuous variables were evaluated for normality. Those conforming to a normal distribution were expressed as mean ± standard deviation, while skewed data were reported as medians with interquartile ranges. Categorical variables were presented as counts and percentages. Between-group differences were assessed using independent samples *t*-tests for normally distributed continuous variables and the Mann–Whitney U test for non-normally distributed data. Categorical comparisons were performed using Pearson’s chi-square test or Fisher’s exact test where appropriate.

To identify potential risk factors for ICT and to screen for predictive inflammation-related biomarkers among AMI patients, univariate logistic regression analyses were conducted. Variables with a *p* < 0.05 in univariate analysis were subsequently entered into a multivariate logistic regression model to identify independent predictors. Results were reported as odds ratios with corresponding 95% confidence intervals.

To further assess the predictive role of NLR in ICT, four hierarchical logistic regression models were constructed with progressive adjustment for potential confounders: Model 1 was unadjusted; Model 2 adjusted for age and sex; Model 3 included additional adjustments for hypertension, diabetes, chronic kidney disease, arrhythmia, and Killip class; and Model 4 further accounted for laboratory indicators including cardiac injury biomarkers, liver function enzymes, lipid profile, renal function parameters, electrolytes, and hemoglobin levels. NLR was also categorized into high and low groups based on the optimal threshold determined via ROC curve analysis, and its predictive performance was evaluated across all models.

Based on multivariate analysis, the strongest predictors—NLR and LDL-C—were used to construct overall and sex-specific risk heatmaps. Model performance was assessed using ROC curves and calibration plots. All statistical analyses were conducted using SPSS version 26.0 (IBM Corp., Armonk, NY, USA) and R version 4.5.0. A two-sided *p*-value of < 0.05 was considered statistically significant.

This retrospective observational study was conducted at the First Affiliated Hospital of Xi’an Jiaotong University (No. 277, West Yanta Road, Xi’an, Shaanxi Province, 710061, China). Clinical data were retrospectively collected from patients diagnosed with acute myocardial infarction (AMI) between January 2018 and December 2022. AMI and ICT were diagnosed according to established criteria (12, 13), and treatment protocols adhered to the 2023 European Society of Cardiology guidelines for the management of acute coronary syndromes (14). Eligible patients included those with confirmed diagnoses of AMI, including both ST-elevation myocardial infarction and non-ST-elevation myocardial infarction. ICT was confirmed through echocardiographic imaging performed during hospitalization, encompassing thrombus detection in the left and right ventricles, atria, atrial appendages, apex, or other cardiac chambers (6, 15).

## 3 Results

### 3.1 Clinical characteristics of study participants

A total of 9,415 patients diagnosed with AMI were admitted to the First Affiliated Hospital of Xi’an Jiaotong University. After excluding 416 patients due to incomplete laboratory data, 8,999 remained eligible for analysis, among whom 88 were diagnosed with ICT. Given the low prevalence of ICT, to address class imbalance and improve model stability and discriminatory performance, a 1:10 ratio of non-ICT controls was randomly sampled, resulting in a final cohort of 979 patients (88 ICT cases and 891 controls). For variables with missing values (<5%), multiple imputation by chained equations (MICE) was performed. The imputation model included all relevant predictors and outcome variables used in the main analysis, such as demographics, comorbidities, Killip classification, and laboratory parameters including inflammatory, cardiac, hepatic, renal, and hematologic indices. To improve transparency, we summarized the missingness for each laboratory covariate and presented their means and standard deviations in Supplementary Table S2. Given the low level of missingness, the distributions of variables before and after imputation were comparable, with no substantial shifts observed. Five imputed datasets were generated and combined using Rubin’s rules to minimize bias and optimize statistical power. No significant differences were observed between groups in baseline demographics, comorbidities, or Killip class. However, several laboratory parameters differed significantly: the ICT group had higher levels of NLR, CK-MB, CK, LDH, AST, ALT, NT-proBNP, cTnT, and LDL-C, while AGR was significantly lower (all *p* < 0.05). Detailed baseline characteristics are presented in Table 1.

**Table 1:** Baseline characteristics of patients with AMI.

Variables	Total (*n* = 979)	Control group (*n* = 891)	ICT group (n = 88)	*p*-value
Enrollment basic characteristics				
Age (years, IQR)	64.00 (55.00, 71.00)	64.00 (55.00, 71.00)	62.00 (55.00, 71.00)	0.986
Female (*n*, %)	253 (25.84)	236 (26.49)	17 (19.32)	0.143
Comorbidities				
Hypertension (*n*, %)	457 (46.68)	415 (46.58)	42 (47.73)	0.837
Diabates (*n*, %)	600 (61.29)	543 (60.94)	57 (64.77)	0.482
CKD (*n*, %)	7 (0.72)	5 (0.56)	2 (2.27)	0.125
Arrhythmia (*n*, %)	82 (8.38)	72 (8.08)	10 (11.36)	0.289
Killip classification (*n*, %)				
I	815 (83.25)	743 (83.39)	72 (81.82)	0.707
II	122 (12.46)	110 (12.35)	12 (13.64)	0.727
III	23 (2.35)	21 (2.36)	2 (2.27)	1.000
IV	19 (1.941)	17 (1.91)	2 (2.27)	1.000
Laboratory results				
NLR (IQR)	2.83 (2.36–3.45)	2.78 (2.30–3.37)	3.63 (3.07–5.34)	<0.001
AGR (IQR)	2.15 (1.40–2.89)	2.19 (1.55–2.90)	1.60 (1.09–2.39)	<0.001
CK-MB (U/L, IQR)	26.00 (14.00–78.00)	26.00 (14.00–76.00)	34.85 (18.00–106.65)	0.031
CK (U/L, IQR)	225.00 (96.0–763.0)	221.0 (95.00–734.00)	257.50 (126.00–1219.00)	0.024
LDH (U/L, IQR)	279.0 (218.00–432.00)	271.0(216.00–415.00)	420.00 (266.50–705.50)	<0.001
AST (U/L, IQR)	42.00 (25.00–97.00)	41.00 (25.00–92.00)	59.00 (31.50–163.00)	0.004
ALT (U/L, IQR)	31.0 (21.00–47.00)	31.00 (21.00–46.00)	39.00 (25.75–54.50)	0.002
NT-proBNP (pg/mL, IQR)	749.40 (266.40–1879)	697.00 (254.05–1715.00)	2210.0(650.1–5071.25)	<0.001
cTnT (ng/mL, IQR)	0.46 (0.08–1.54)	0.42 (0.07–1.46)	0.89 (0.19–2.48)	<0.001
LDL-C (mmol/L, IQR)	2.13 (1.69–2.70)	2.09 (1.63–2.60)	2.70 (2.27–3.17)	<0.001
HDL-C (mmol/L, IQR)	0.95 (0.81–1.09)	0.95 (0.82–1.10)	0.91 (0.76–1.06)	0.104
BUN (mmol/L, IQR)	5.57 (4.48–6.92)	5.54 (4.46–6.91)	5.72 (4.57–6.93)	0.465
Creatinine (μmol/L, IQR)	64.00 (54.00–77.50)	64.00 (54.00–78.00)	62.50 (53.00–77.00)	0.758
ANC (×10^9^/L, IQR)	6.54 (4.76–9.06)	6.61 (4.79–9.03)	6.05 (4.34–9.12)	0.443
ALC (×10^9^/L, IQR)	1.42 (1.04–1.89)	1.42 (1.04–1.89)	1.43 (1.09–1.90)	0.697
WBC (×10^9^/L, IQR)	8.62 (6.96–11.20)	8.68 (6.99–11.16)	8.22 (6.75–11.38)	0.481
NEUT (100%, IQR)	76.40 (68.40–83.20)	76.40 (68.55–83.30)	76.65 (66.90–81.03)	0.269
Hb (g/L, IQR)	142.00 (129.00–153.50)	142.00 (129.50–154.00)	139.00 (123.25–151.00)	0.123
K^+^ (mmol/L, IQR)	3.95 (3.67–4.23)	3.95 (3.67–4.23)	3.99 (3.66–4.25)	0.707
Na^+^ (mmol/L, IQR)	140.00 (138.00–142.00)	140.00 (138.00–142.00)	139.93 (137.45–142.00)	0.331
Cl^−^ (mmol/L, IQR)	102.00 (99.40–104.90)	102.10 (99.40–104.80)	101.80 (100.00–105.40)	0.618
Ca^2+^ (mmol/L, IQR)	2.26 (2.15–2.35)	2.26 (2.15–2.35)	2.24 (2.11–2.36)	0.320
Mg^2+^ (mmol/L, IQR)	0.98 (0.92–1.06)	0.98 (0.92–1.06)	0.99 (0.91–1.08)	0.870

AMI, acute myocardial infarction; ICT, intracardiac thrombosis; IQR, interquartile range; CKD, chronic kidney disease; NLR, neutrophil-to-lymphocyte ratio; AGR, albumin-to-globulin ratio; CK-MB, creatine kinase–MB isoenzyme; CK, creatine kinase; LDH, lactate dehydrogenase; AST, aspartate aminotransferase; ALT, alanine aminotransferase; NT-proBNP, N-terminal pro–B-type natriuretic peptide; cTnT, cardiac troponin T; LDL-C, low-density lipoprotein cholesterol; HDL-C, high-density lipoprotein cholesterol; BUN, blood urea nitrogen; ANC, absolute neutrophil count; ALC, absolute lymphocyte count; WBC, white blood cell count; NEUT, neutrophil percentage; Hb, hemoglobin; K^+^, serum potassium; Na^+^, serum sodium; Cl^−^, serum chloride; Ca^2+^, serum calcium; Mg^2+^, serum magnesium. All *p*-values presented are unadjusted due to the hypothesis-driven nature of the analysis; multivariable models were used to confirm independent associations.

### 3.2 Identification of inflammation-related predictors of ICT

Baseline demographic and clinical variables were compared between the ICT and non-ICT groups. NLR, CK-MB, CK, LDH, ALT, NT-proBNP, cTnT, LDL-C, and AGR were significantly associated with ICT (*p* < 0.05). These variables were entered into multivariate logistic regression (Table 2), identifying NLR [odds ratio (OR) = 2.25, 95% confidence interval (CI): 1.79–2.83, *p* < 0.001], NT-proBNP (OR = 1.01, 95% CI: 1.00–1.02, *p* = 0.012), and LDL-C (OR = 2.86, 95% CI: 2.03–4.02, *p* < 0.001) as independent predictors, while AGR was protective (OR = 0.79, 95% CI: 0.64–0.97, *p* = 0.026).

**Table 2:** Results of multivariate logistic regression model.

Variables	Univariate regression (*p* < 0.05)		Multivariate analysis	
	OR (95% CI)	*p*-value	OR (95% CI)	*p*-value
NLR	2.35 (1.90–2.90)	<0.001	**2.25 (1.79–2.83)**	**<0.001**
AGR	0.67 (0.53–0.84)	<0.001	**0.79 (0.64–0.97)**	**0.026**
CK-MB (U/L)	1.01 (1.00–1.03)	<0.001	1.00 (1.00–1.01)	0.709
CK (U/L)	1.05 (1.03–1.07)	<0.001	1.00 (1.00–1.00)	0.871
LDH (U/L)	1.01 (1.01–1.01)	<0.001	1.00 (1.00–1.00)	0.131
AST (U/L)	1.01 (1.01–1.01)	<0.001	1.00 (0.99–1.00)	0.162
ALT (U/L)	1.00 (1.00–1.00)	0.078		
NT-proBNP (pg/mL)	1.02 (1.00–1.04)	<0.001	**1.01 (1.00–1.02)**	**0.012**
cTnT (ng/mL)	1.15 (1.06–1.24)	<0.001	1.07 (0.92–1.24)	0.371
LDL-C (mmol/L)	2.75 (2.06–3.67)	<0.001	**2.86 (2.03–4.02)**	**<0.001**

NLR, neutrophil-to-lymphocyte ratio; AGR, albumin-to-globulin ratio; CK-MB, creatine kinase–MB isoenzyme; CK, creatine kinase; LDH, lactate dehydrogenase; AST, aspartate aminotransferase; NT-proBNP, N-terminal pro–B-type natriuretic peptide; cTnT, cardiac troponin T; LDL-C, low-density lipoprotein cholesterol. Bold values indicate statistical significance (*p* < 0.05) in the multivariate logistic regression analysis.

**Figure 2:**
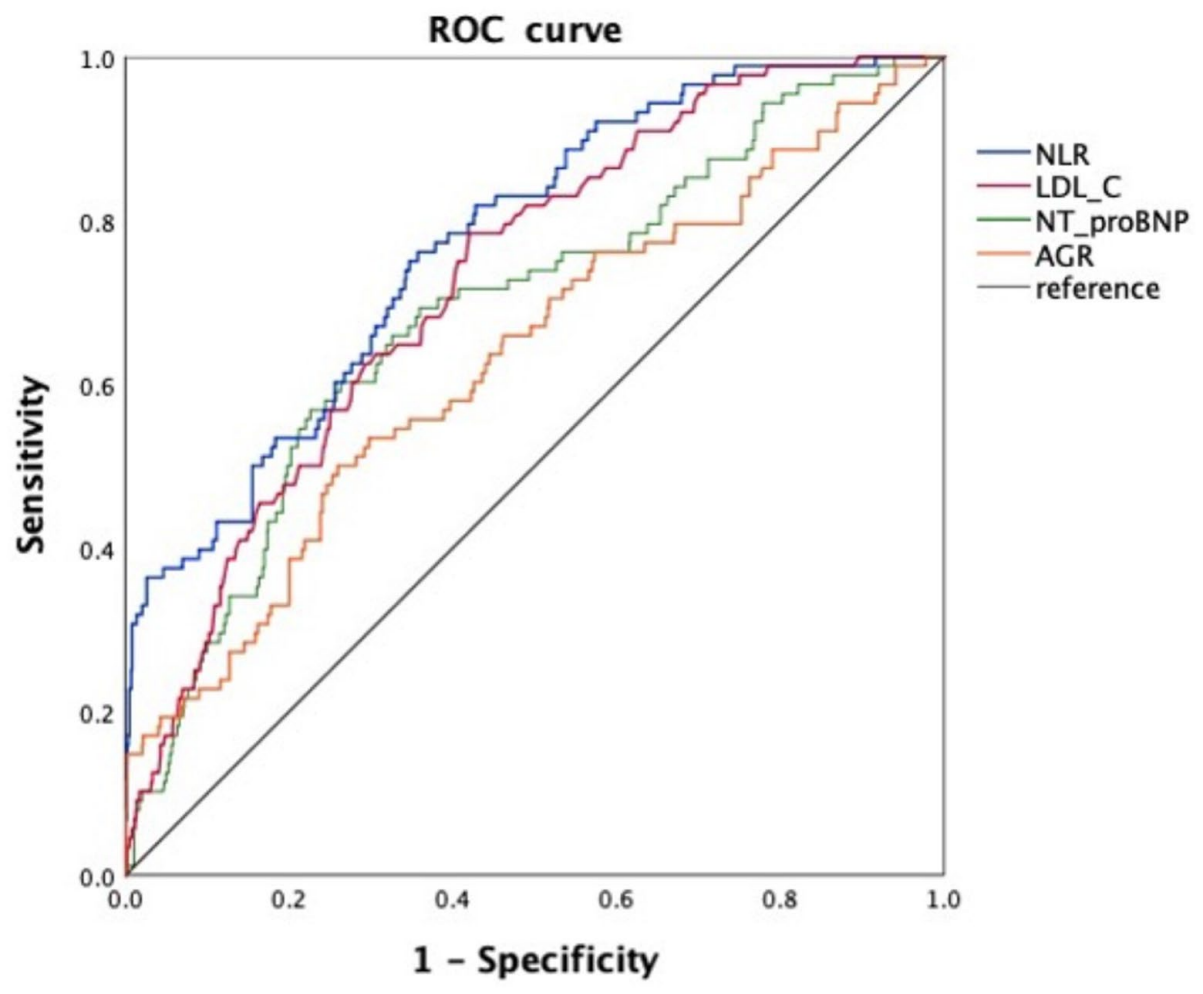
ROC curves of four inflammation-related biomarkers for predicting ICT in patients with AMI. NLR, neutrophil-to-lymphocyte ratio; LDL-C, low-density lipoprotein cholesterol; NT-proBNP, N-terminal pro–B-type natriuretic peptide; AGR, albumin-to-globulin ratio.

Receiver operating characteristic (ROC) curve analysis was performed to evaluate the predictive performance of these markers (Figure 2). NLR [area under the curve (AUC) = 0.774, 95% CI: 0.724–0.823, *p* < 0.001] and LDL-C (AUC = 0.728, 95% CI: 0.677–0.778, *p* < 0.001) demonstrated favorable discriminative ability. These findings highlight the potential clinical utility of NLR and LDL-C as inflammation-related biomarkers for predicting ICT in AMI patients.

### 3.3 Predictive value of NLR after adjustment and stratification

To evaluate the independent predictive value of NLR for ICT, we constructed four logistic regression models with progressive adjustment for potential confounders.

**Figure 3:**
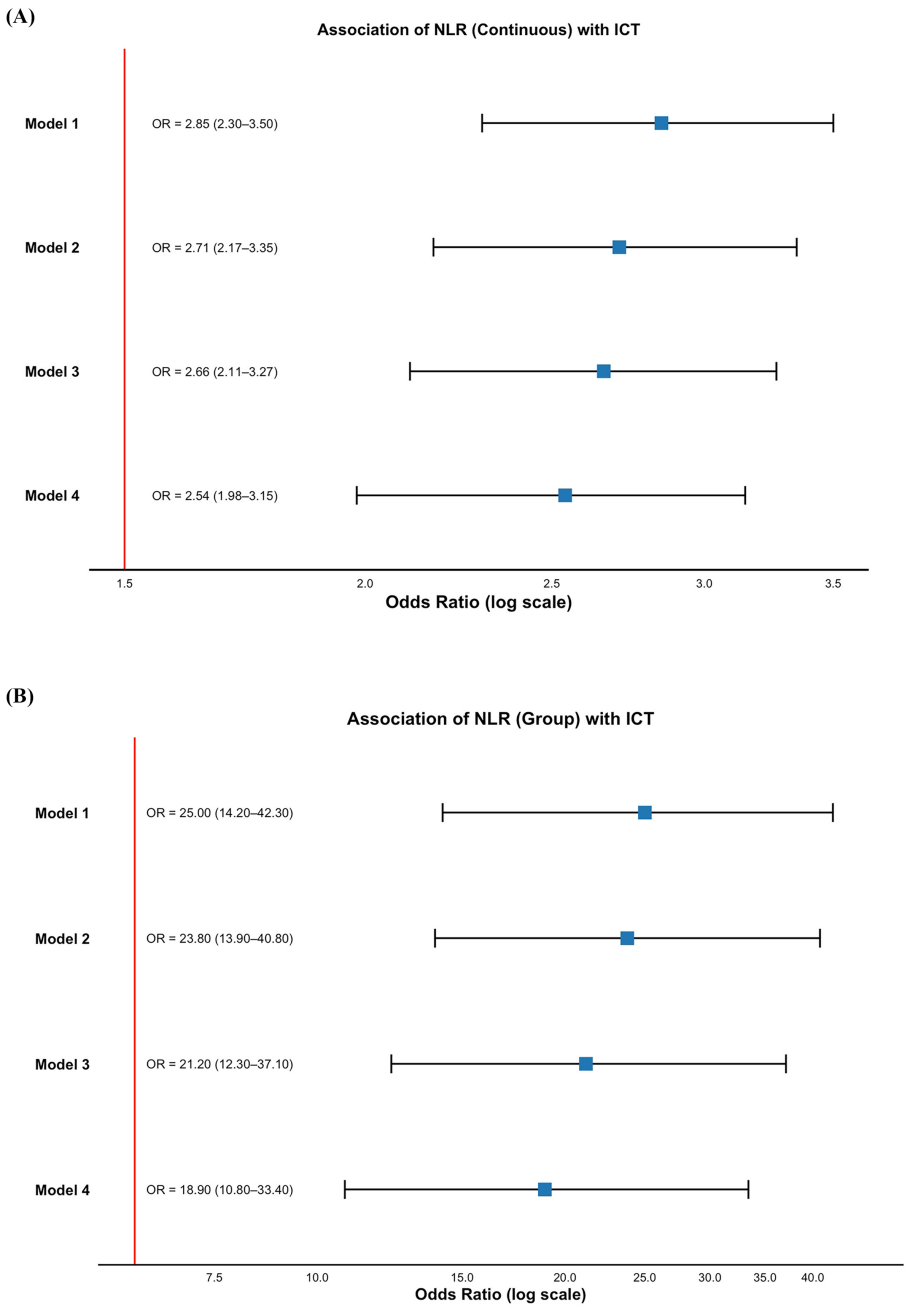
Association between NLR and ICT in patients with AMI. **(A)** NLR as a continuous variable; **(B)** NLR as a categorical variable. Model 1: unadjusted; Model 2: adjusted for age and sex; Model 3: adjusted for age, sex, comorbidities (hypertension, diabetes, CKD, arrhythmia), and Killip classification; Model 4: additionally adjusted for laboratory parameters including liver function, renal function, serum electrolytes, and cardiac injury biomarkers. AMI, acute myocardial infarction; ICT, intracardiac thrombosis; NLR, neutrophil-to-lymphocyte ratio; CKD, chronic kidney disease; OR, odds ratio; CI, confidence interval.

When included as a continuous variable, NLR remained significantly associated with ICT across all four models (all *p* < 0.001), demonstrating consistent independent predictive value. In the fully adjusted Model 4, the OR for NLR was 2.54 (95% CI: 1.98–3.15) (Figure 3A). We then stratified NLR using the ROC-derived cut-off value of 3.894 and analyzed it as a categorical variable. NLR remained significantly predictive of ICT in all models, with an OR of 18.9 (95% CI: 10.8–33.4) in Model 4 (Figure 3B), further supporting its role as a stable and reliable inflammation-related biomarker for predicting ICT in AMI patients.

### 3.4 Visual heatmap model for risk prediction

**Figure 4:**
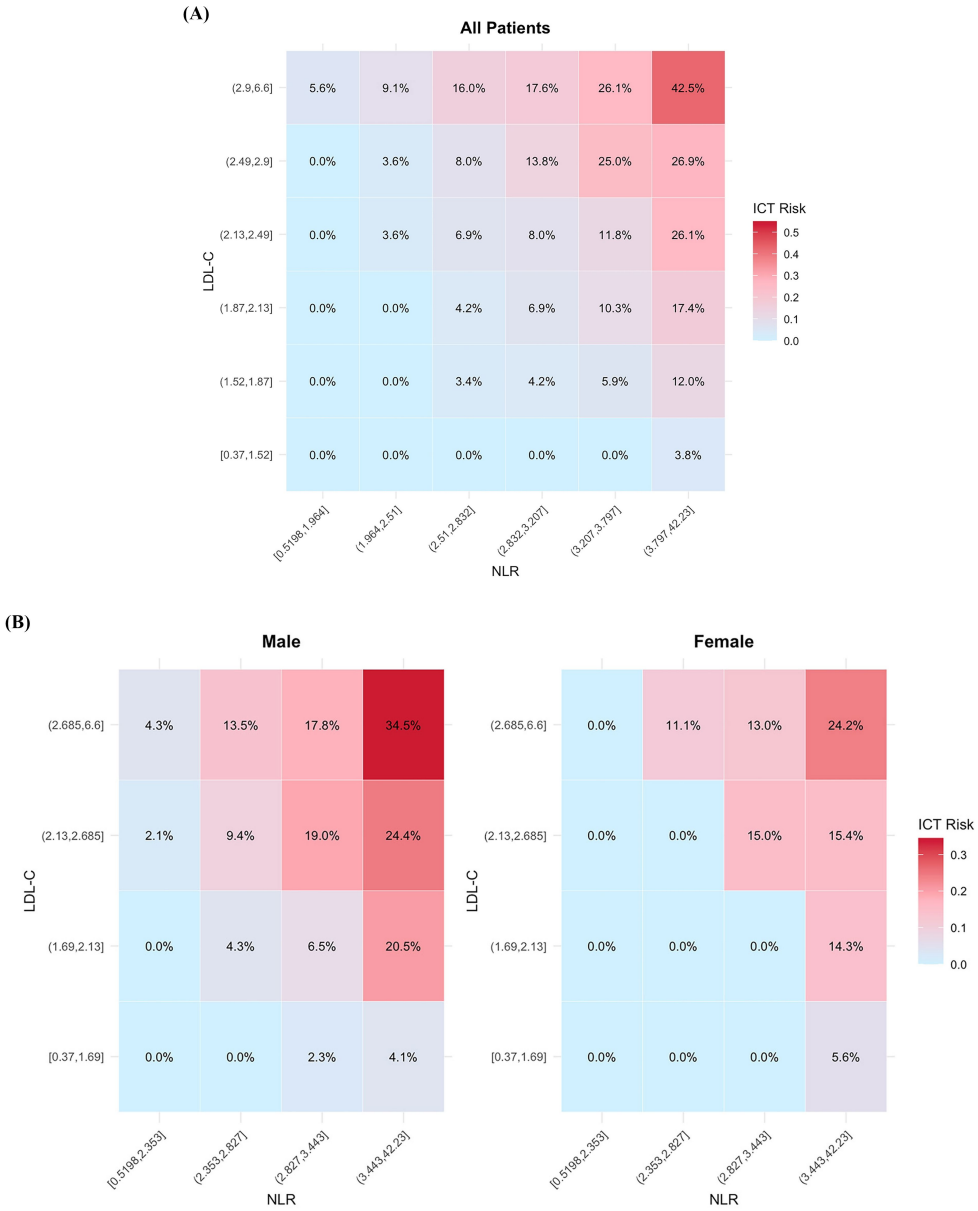
Heatmap visualization of ICT risk based on the combined distribution of NLR and LDL-C. **(A)** Risk heatmap for the overall cohort. **(B)** Sex-stratified risk heatmaps. ICT, intracardiac thrombosis; NLR, neutrophil-to-lymphocyte ratio; LDL-C, low-density lipoprotein cholesterol.

To enhance the clinical applicability of our findings, we developed a visual risk heatmap model based on the two top-performing inflammation-related biomarkers—NLR and LDL-C. Both markers were divided into sextiles, and a combined risk heatmap was generated for the entire cohort (Figure 4A), followed by sex-stratified heatmaps to visualize differential risk distribution (Figure 4B). This model intuitively illustrated the probability of ICT occurrence across varying levels of NLR and LDL-C.

**Figure 5:**
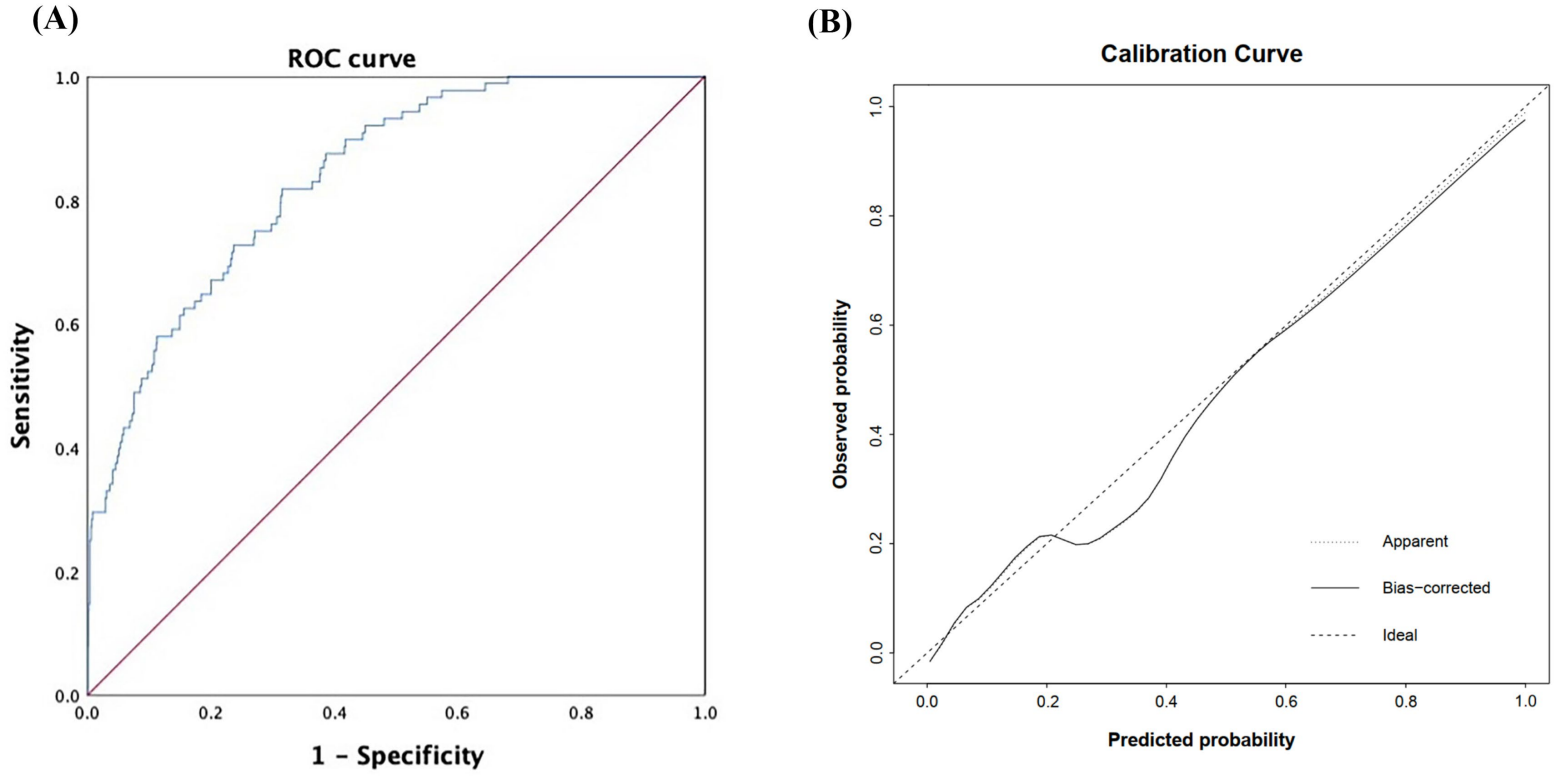
Discrimination and calibration performance of the risk heatmap model based on NLR and LDL-C. **(A)** ROC curve of the model **(B)** calibration curve assessing the agreement between predicted and observed probabilities. NLR, neutrophil-to-lymphocyte ratio; LDL-C, low-density lipoprotein cholesterol; ROC, receiver operating characteristic; AUC, area under the curve.

The model achieved strong discriminatory performance with an AUC of 0.838 (95% CI: 0.799–0.878), outperforming either marker alone (Figure 5A). The calibration curve showed close agreement between predicted and observed probabilities, and the Hosmer–Lemeshow test (*χ*^2^ = 8.872, *p* = 0.357) confirmed the model’s goodness of fit and reliability (Figure 5B).

## 4 Discussion

This study investigated the predictive value of inflammation-related biomarkers for ICT in patients with AMI. The main findings are as follows: (1) four biomarkers—elevated NLR, LDL-C, NT-proBNP, and decreased AGR—were independently associated with ICT occurrence in multivariable logistic regression (all *p* < 0.05); (2) among them, NLR demonstrated the strongest predictive power, remaining statistically significant across all adjusted models (fully adjusted OR = 2.54; subgroup OR = 18.9); (3) a risk heatmap combining NLR and LDL-C exhibited good discriminatory performance (AUC = 0.838), suggesting its potential clinical utility in ICT risk stratification in AMI patients.

Inflammation plays a central role in AMI, not only as a response to cardiomyocyte necrosis but also as a driver of post-infarction complications. Necrotic myocardium releases cytokines such as IL-6, TNF-*α*, and IL-1β, which recruit neutrophils and monocytes to the infarcted area (16). This inflammatory cascade may aggravate injury, trigger oxidative stress, and activate coagulation, fostering a prothrombotic state (17, 18). Persistent inflammation following AMI contributes to endothelial dysfunction, tissue factor expression, and platelet activation, all of which promote ICT (19). As noted by Frangogiannis, inflammation orchestrates both early tissue damage and late-stage thrombus deposition (20). Structural substrates, including anterior wall infarction, reduced LVEF, ventricular aneurysm, and high Killip class, are established risk factors for left ventricular thrombus (LVT) (21–23), and elevated CK-MB levels are also associated with thrombosis (24). Although Sia et al. linked elevated NLR with LVT (25), data on inflammation-based predictors for other ICT subtypes remain limited. Additionally, neutrophil extracellular traps (NETs) contribute to thrombogenesis by interacting with TF and platelets within infarcted myocardium (25, 26), suggesting inflammation may directly participate in ICT pathogenesis.

The NLR is a simple and accessible inflammation-related biomarker derived from routine peripheral blood counts, reflecting the balance between innate immune activation and adaptive immune suppression (27). In our study, NLR was the strongest independent predictor of intracardiac thrombosis (ICT) among all evaluated biomarkers, with its predictive value remaining robust across fully adjusted and subgroup models. Previous studies have linked elevated NLR to adverse cardiovascular outcomes, including acute coronary syndrome, heart failure, and stroke (28–30). While Zazula et al. demonstrated its association with left ventricular thrombus (LVT) in AMI patients (31), more recent findings suggest that elevated NLR is also associated with left atrial thrombus and spontaneous echocardiographic contrast in patients with atrial fibrillation or dilated ventricles (32, 33), supporting its broader role in identifying prothrombotic conditions across cardiac chambers. Mechanistically, an elevated NLR reflects a shift toward a pro-inflammatory and pro-thrombotic state. Activated neutrophils release reactive oxygen species, proteolytic enzymes, and myeloperoxidase, which contribute to endothelial damage and upregulate tissue factor expression (34). In addition, neutrophil extracellular traps (NETs)—web-like DNA structures enriched with tissue factor and platelets—facilitate thrombus propagation (35–37). Concurrently, reduced lymphocyte levels indicate impaired immunoregulation, promoting sustained neutrophil activation and thrombin generation (38). These immuno-thrombotic mechanisms provide a biological rationale for the association between elevated NLR and ICT development (26, 39). In our cohort, an NLR threshold of 3.894 was derived using receiver operating characteristic (ROC) analysis, offering an optimal trade-off between sensitivity and specificity for ICT risk stratification. Although cut-off values may vary across populations, similar thresholds have been reported—for example, 4.25 for predicting ventricular remodeling following anterior STEMI (40), and 5.509 for in-hospital mortality in NSTEMI patients (41). Accordingly, we applied this ROC-derived threshold to facilitate stratified analysis and improve clinical interpretability, while acknowledging that it is context-specific and requires external validation in independent cohorts.

Our study identified elevated low-density lipoprotein cholesterol (LDL-C) as an independent predictor of ICT in patients with AMI, with significant predictive value (OR = 2.86, 95% CI: 2.03–4.02, *p* < 0.001). Mechanistically, LDL-C contributes to post-AMI thrombogenesis through multiple pathways. Oxidized LDL promotes endothelial expression of tissue factor and adhesion molecules, thereby activating the factor VII–dependent extrinsic coagulation cascade and facilitating thrombus formation (42). It also upregulates inflammatory mediators such as MCP-1 and IL-6, enhancing local inflammation and plaque instability (43). In parallel, LDL-C binds to the platelet surface receptor CD36, promoting platelet adhesion and aggregation (44), which in the prothrombotic milieu of AMI further amplifies clot formation. Moreover, elevated LDL-C is closely associated with endothelial dysfunction. It reduces nitric oxide (NO) bioavailability and impairs anti-inflammatory and antiplatelet responses, while abnormal vascular tone promotes local flow disturbances that favor thrombus development (44, 45). Collectively, these findings suggest that beyond its role in atherogenesis, LDL-C may actively drive ICT via a cascade involving inflammation, endothelial injury, and coagulation activation. As such, LDL-C represents a clinically relevant inflammation-related biomarker for ICT risk stratification in AMI patients (46).

To improve bedside applicability, we further incorporated NLR and LDL-C into a visual risk heatmap, enabling intuitive stratification of ICT risk using admission laboratory data. This tool may assist clinicians in identifying AMI patients at heightened thrombotic risk who warrant early or repeat echocardiographic screening—even in the absence of overt clinical signs. In scenarios with limited imaging availability or diagnostic uncertainty, the heatmap may also support individualized decisions regarding closer surveillance or early initiation of prophylactic anticoagulation, in accordance with bleeding risk profiles. By translating biomarker data into a user-friendly format, this approach provides a practical aid for timely and personalized decision-making in routine AMI care.

In addition to NLR and LDL-C, our analysis identified elevated NT-proBNP and decreased AGR as independent predictors of ICT in AMI patients. NT-proBNP, a marker of myocardial stress and volume overload, may reflect left ventricular dilation and intracardiac stasis—conditions that favor thrombus formation. Prior studies have linked NT-proBNP levels to increased risk of left ventricular thrombus in anterior STEMI patients (47–49). AGR, an index reflecting systemic inflammation and nutritional status, has been associated with adverse cardiovascular outcomes and in-hospital mortality (50, 51). Mechanistically, a reduced AGR may indicate persistent inflammation and impaired immune regulation, thereby promoting endothelial dysfunction and coagulation activation (52). Together, these markers may provide supplementary predictive value beyond traditional inflammatory and lipid indicators.

We also observed that male patients had a higher risk of ICT than females at similar levels of inflammatory biomarkers, suggesting a possible sex-based vulnerability. While the underlying mechanisms remain unclear, prior studies have reported sex-specific differences in thrombotic susceptibility, vascular response, and diagnostic patterns in AMI patients (53–55). These findings warrant further investigation in sex-stratified prospective cohorts.

We acknowledge several limitations in this study. Firstly, the retrospective nature of data collection introduces a potential risk of information bias, such as incomplete clinical documentation and variability in diagnostic testing. These factors may compromise the accuracy of outcome ascertainment and the consistency of biomarker evaluation. Moreover, the retrospective design inherently limits causal inference between inflammation-related biomarkers and clinical outcomes.

Second, there is a risk of residual confounding due to unmeasured or incompletely captured clinical variables, such as medication use (e.g., anticoagulants) and in-hospital treatments (e.g., thrombolysis, percutaneous interventions). These factors may influence both systemic inflammation and thrombosis risk but were not adequately adjusted for due to dataset limitations. Third, the incidence of ICT observed in our study was 1%, which is notably lower than the 5–8% reported in earlier literature (56, 57). This discrepancy may be attributed to several factors, including the absence of recent large-scale epidemiological data, improvements in clinical management that have reduced thrombotic complications, and the early and effective interventions available at our center, a high-volume tertiary hospital in Northwestern China. Lastly, while we applied a 1:10 random sampling strategy to address class imbalance, this approach may have introduced information loss or sampling variability, potentially affecting model robustness. Despite these limitations, our study provides important evidence on the role of inflammation-related biomarkers in predicting ICT among patients with acute myocardial infarction. It helps to address a current gap in the literature and lays the groundwork for improved risk stratification and individualized management in clinical practice.

## 5 Conclusion

In this large retrospective analysis of 8,999 patients with AMI, we employed a 1:10 case–control matching strategy to systematically evaluate the predictive value of inflammation-related biomarkers for ICT. NLR, NT-proBNP, and LDL-C were identified as independent risk factors, while AGR appeared protective. Multivariable logistic regression and sensitivity analyses consistently confirmed the robust predictive power of NLR, which remained significant even after full adjustment (OR = 18.9). Building on these findings, we developed a visual risk heatmap model based on NLR and LDL-C, which demonstrated strong discrimination (AUC = 0.838) and good calibration, thereby enhancing clinical applicability. Our findings provide a practical tool for early ICT risk stratification in AMI patients, underscore the pivotal role of inflammation in post-AMI thrombogenesis, and offer new perspectives on the application of inflammatory biomarkers in cardiovascular disease management.

## Data Availability

The datasets presented in this article are not readily available because raw data cannot be provided due to laboratory policies and confidentiality agreements. However, we are available for any inquiries or further clarification if needed. Requests to access the datasets should be directed to NM, mn19761221@mail.xjtu.edu.cn.
