# Supplementary material for: Inflammation-related biomarkers for intracardiac thrombosis in acute myocardial infarction: predictive value and mechanistic implications of NLR and LDL-C
Source: Front Med (Lausanne). 2025 Aug 5;12:1643933. doi: 10.3389/fmed.2025.1643933 (PMC12361238; doi:10.3389/fmed.2025.1643933)
Supplement: Supplementary file 1 [file Table_1.pdf]

Supplementary Table 1. Comparison of Baseline Characteristics Between Included and Excluded Non-ICT AMI Patients

Variables	Total (n = 8911)	Included (n = 891)	Excluded (n = 8020)	*p*-Value
**Enrollment basic characteristics**				
Age (years, IQR)	63.06 ± 11.96	62.91 ± 11.02	63.08 ± 12.06	0.667
Female (n, %)	2494 (27.99)	236 (26.49)	2258 (28.15)	0.293
**Comorbidities**				
Hypertension (n, %)	4148 (46.55)	415 (46.58)	3733 (46.55)	0.764
Diabates (n, %)	5673 (63.66)	543 (61.94)	5130 (63.97)	0.088
CKD (n, %)	79 (0.89)	5 (0.56)	74 (0.92)	0.275
Arrhythmia (n, %)	722 (8.10)	72 (8.08)	650 (8.10)	0.680
**Killip classification (n, %)**				
I	7560 (84.84)	743 (83.39)	6817 (84.95)	0.380
II	979 (10.99)	110 (12.35)	869 (10.84)	0.304
III	181 (2.03)	21 (2.36)	160 (2.00)	0.634
IV	191 (2.14)	17 (1.91)	174 (2.17)	0.609
**Laboratory results**				
NLR (mean ± SD)	2.62 ± 0.73	2.64 ± 0.69	2.64 ± 0.71	0.908
AGR (mean ± SD)	2.76 ± 0.90	2.76 ± 0.84	2.75 ± 1.28	0.697
CK-MB (U/L, mean ± SD)	76.98 ± 124.96	72.76 ± 117.14	77.48 ± 125.86	0.286
CK (U/L, mean ± SD)	753.93 ± 1421.47	700.02 ± 1243.14	760.24 ± 1440.82	0.232

LDH (U/L, mean ± SD)	375.27 ± 309.31	368.23 ± 313.28	376.09 ± 308.85	0.473
AST (U/L, mean ± SD)	91.07 ± 147.39	85.18 ± 131.47	91.78 ± 149.17	0.207
ALT (U/L, mean ± SD)	44.16 ± 99.16	40.62 ± 69.64	44.59 ± 102.22	0.259
NT-proBNP (pg/mL, mean ± SD)	1875.42 ± 4091.07	1865.46 ± 4064.68	1876.63 ± 4094.55	0.939
cTnT (ng/mL, mean ± SD)	1.29 ± 2.13	1.21 ± 2.09	1.30 ± 2.14	0.290
LDL-C (mmol/L, mean ± SD)	2.18 ± 0.81	2.16 ± 0.71	2.18 ± 0.82	0.634
HDL-C (mmol/L, mean ± SD)	0.98 ± 0.23	0.97 ± 0.22	0.98 ± 0.23	0.925
BUN (mmol/L, mean ± SD)	6.05 ± 2.73	6.13 ± 2.98	6.04 ± 2.70	0.344
Creatinine (μmol/L, mean ± SD)	74.14 ± 61.05	73.10 ± 53.98	74.26 ± 61.82	0.593
ANC (×109/L, mean ± SD)	7.33 ± 3.49	7.23 ± 3.28	7.34 ± 3.51	0.374
ALC (×109/L, mean ± SD)	1.53 ± 0.71	1.52 ± 0.67	1.53 ± 0.71	0.718
WBC (×109/L, mean ± SD)	9.46 ± 3.61	9.36 ± 3.39	9.47 ± 3.63	0.343
NEUT (100%, mean ± SD)	75.36 ± 10.33	75.28 ± 10.17	75.36 ± 10.35	0.829
Hb (g/L, mean ± SD)	140.23 ± 18.79	140.64 ± 19.29	140.19 ± 18.74	0.496
K^+ (mmol/L, IQR)	3.95 (3.67, 4.24)	3.95 (3.67, 4.23)	3.96 (3.67, 4.24)	0.548
Na^+ (mmol/L, mean ± SD)	139.90 ± 3.61	139.93 ± 3.69	139.90 ± 3.60	0.786
Cl^- (mmol/L,IQR)	102.00 (99.30, 104.80)	102.00 (99.20, 104.75)	102.00 (99.30, 104.80)	0.601
Ca^{2+} (mmol/L, mean ± SD)	2.24 ± 0.16	2.25 ± 0.16	2.24 ± 0.16	0.215
Mg^{2+} (mmol/L, mean ± SD)	0.99 ± 0.13	1.00 ± 0.13	0.99 ± 0.13	0.153

* AMI, acute myocardial infarction; ICT, intracardiac thrombosis; IQR, interquartile range; SD, standard deviation; CKD, chronic kidney disease; NLR, neutrophil-to-lymphocyte ratio; AGR,

albumin-to-globulin ratio; CK-MB, creatine kinase–MB isoenzyme; CK, creatine kinase; LDH, lactate dehydrogenase; AST, aspartate aminotransferase; ALT, alanine aminotransferase; NT-proBNP, N-terminal pro–B-type natriuretic peptide; cTnT, cardiac troponin T; LDL-C, low-density lipoprotein cholesterol; HDL-C, high-density lipoprotein cholesterol; BUN, blood urea nitrogen; ANC, absolute neutrophil count; ALC, absolute lymphocyte count; WBC, white blood cell count; NEUT, neutrophil percentage; Hb, hemoglobin; K^+, serum potassium; Na^+, serum sodium; Cl^-, serum chloride; Ca^{2+}, serum calcium; Mg^{2+}, serum magnesium.

"Included" refers to the non-ICT AMI patients (n = 891) randomly selected as controls in the main analysis. "Excluded" refers to the remaining AMI patients without ICT (n = 8,020) who were not included in the final analytic sample.
