# Supplementary material for: Inflammation-related biomarkers for intracardiac thrombosis in acute myocardial infarction: predictive value and mechanistic implications of NLR and LDL-C
Source: Front Med (Lausanne). 2025 Aug 5;12:1643933. doi: 10.3389/fmed.2025.1643933 (PMC12361238; doi:10.3389/fmed.2025.1643933)
Supplement: Supplementary file 2 [file Table_2.docx]

**Supplementary Table S2. Summary statistics and missingness for laboratory variables included in multiple imputation**

| **Variables** | **n** | **Mean** | **SD** | **Missing (n)** | **Missing (%)** |
| --- | --- | --- | --- | --- | --- |
| AGR | 975 | 2.73 | 0.82 | 4 | 0.4% |
| CK-MB(U/L) | 977 | 72.18 | 117.70 | 2 | 0.2% |
| ALT(U/L) | 972 | 41.14 | 94.10 | 7 | 0.7% |
| NT-proBNP (pg/mL) | 966 | 1866.81 | 4061.77 | 13 | 1.3% |
| LDL-C(mmol/L) | 975 | 2.16 | 0.71 | 4 | 0.4% |
| HDL-C(mmol/L) | 975 | 0.97 | 0.22 | 4 | 0.4% |
| BUN (mmol/L) | 978 | 6.13 | 2.98 | 1 | 0.1% |
| Creatine(μmol/L) | 978 | 73.10 | 53.96 | 1 | 0.1% |

*AGR, albumin-to-globulin ratio; CK-MB, creatine kinase-MB isoenzyme; ALT, alanine aminotransferase; NT-proBNP, N-terminal pro–B-type natriuretic peptide; LDL-C, low-density lipoprotein cholesterol; HDL-C, high-density lipoprotein cholesterol; BUN, blood urea nitrogen

This table summarizes the central tendency, dispersion, and missingness of key laboratory variables prior to multiple imputation. All variables with missing values below 5% were imputed using the MICE algorithm.
